# Spatial ecology of moose in Sweden: Combined Sr-O-C isotope analyses of bone and antler

**DOI:** 10.1371/journal.pone.0300867

**Published:** 2024-04-10

**Authors:** Elena Armaroli, Federico Lugli, Anna Cipriani, Thomas Tütken

**Affiliations:** 1 Department of Chemical and Geological Sciences, University of Modena and Reggio Emilia, Modena, Italy; 2 Institut für Geowissenschaften, Goethe Universität Frankfurt, Frankfurt am Main, Germany; 3 Department of Cultural Heritage, University of Bologna, Ravenna, Italy; 4 Lamont-Doherty Earth Observatory, Columbia University, Palisades, NY, United States of America; 5 Arbeitsgruppe für Angewandte und Analytische Paläontologie, Institut für Geowissenschaften, Johannes Gutenberg–Universität Mainz, Mainz, Germany; The Cyprus Institute, CYPRUS

## Abstract

The study of spatial (paleo)ecology in mammals is critical to understand how animals adapt to and exploit their environment. In this work we analysed the ^87^Sr/^86^Sr, δ^18^O and δ^13^C isotope composition of 65 moose bone and antler samples from Sweden from wild-shot individuals dated between 1800 and 1994 to study moose mobility and feeding behaviour for (paleo)ecological applications. Sr data were compared with isoscapes of the Scandinavian region, built *ad-hoc* during this study, to understand how moose utilise the landscape in Northern Europe. The ^87^Sr/^86^Sr isoscape was developed using a machine-learning approach with external geo-environmental predictors and literature data. Similarly, a δ^18^O isoscape, obtained from average annual precipitation δ^18^O values, was employed to highlight differences in the isotope composition of the local environment vs. bone/antler. Overall, 82% of the moose samples were compatible with the likely local isotope composition (n = 53), suggesting that they were shot not far from their year-round dwelling area. ‘Local’ samples were used to calibrate the two isoscapes, to improve the prediction of provenance for the presumably ‘non-local’ individuals. For the latter (n = 12, of which two are antlers and ten are bones), the probability of geographic origin was estimated using a Bayesian approach by combining the two isoscapes. Interestingly, two of these samples (one antler and one bone) seem to come from areas more than 250 km away from the place where the animals were hunted, indicating a possible remarkable intra-annual mobility. Finally, the δ^13^C data were compared with the forest cover of Sweden and ultimately used to understand the dietary preference of moose. We interpreted a difference in δ^13^C values of antlers (^13^C-enriched) and bones (^13^C-depleted) as a joint effect of seasonal variations in moose diet and, possibly, physiological stresses during winter-time, i.e., increased consumption of endogenous ^13^C-depleted lipids.

## 1. Introduction

The study of species dynamics through space and time, including their movements, feeding behaviour, interactions and responses to environmental and anthropogenic factors, constitutes the complex field of spatial ecology [1, 2]. This discipline is increasingly applied to conservation and management issues to find effective ways to preserve biodiversity in an era of profound environmental change [3–6]. In fact, the changing climate and its consequences (e.g., droughts, wildfires, floods, hurricanes), further accelerated by human activities, are causing disturbance of natural habitats in increasingly unpredictable ways. Therefore, it has become important to understand how species respond to our changing world and implement ways to protect our biodiversity [7, 8].

The first step in enabling biodiversity and ecosystem preservation is to study how wildlife species move across the landscape and to quantify and predict their spatial distribution [9, 10]. Today this is possible through an integrative approach that brings together ecological theory and statistical modelling [11–14]. At the same time, to interpret present and future environmental changes it is important to understand what changes have occurred over time. This requires more multidisciplinary research, enabling collaboration among archaeologists, historians, and natural scientists [15–17].

Animal movements are commonly reconstructed by satellite imaging/GPS tracking [18], yet this approach requires marking or recapturing individuals, precluding retrospective investigations from sampled tissues and from archaeological/paleontological specimens. In this sense, biogeochemical markers can help unravel animal life-histories and migratory behaviour. Indeed, geochemistry has long been used for provenance and environmental studies, with applications ranging from food science to ecology, forensics and archaeology [19–23]. It is now well established that mobility and nutritional patterns are recorded in the geochemical signature of human and animal skeletal tissues [24–26]. To date, numerous applications of strontium (^87^Sr/^86^Sr), oxygen (δ^18^O) and carbon (δ^13^C) isotope analyses have reconstructed dietary habits, migration events, residential patterns, animal management, exchange of goods and raw materials in archaeological contexts [27–30].

Studies on modern animals’ spatial ecology are key in understanding how isotopes are linked with individual mobility. This is due to the fact that modern animal behaviour is known and can be compared with findings obtained through geochemical analyses [31]. Moreover, modern samples are not affected by diagenetic modification of bone bioapatite, avoiding the problems of obtaining pristine isotopic compositions [32, 33]. Altogether, modern tissues are thus ideal control samples for calibrating our inferences about past mobility from fossil specimens [34, 35].

A key aspect in provenance studies that use isotope analyses, is the determination of bioavailable Sr in the area of interest to determine whether the individual or object under investigation is local or not [36, 37]. However, this is not sufficient to determine where they came from. In recent years much attention has been focused on the construction of both local and global isoscapes (i.e. isotope distribution maps) to enable the prediction of the place of origin of unknown samples using both Sr and O isotope data [38–40].

Here we used modern and historical *Alces alces* (Linnaeus, 1758) samples (time range 1800–1994) from Sweden to understand the potential of isotope markers in unraveling moose home-range and migratory behaviour. To this end, we measured Sr, O and C in moose bones and antlers and compared their values with a novel built isoscape of the Scandinavian region.

Moose is the largest member of the Cervidae family, distributed in the circumpolar boreal forests of Eurasia and North America [41, 42]. The size of moose populations and their distribution in Europe has changed over time [43–45]. Today moose are widely distributed in Scandinavia and are the dominant large herbivore in Sweden. This animal is currently the focus of an increasing amount of research on its management and conservation [46, 47]. Monitoring moose behaviour has proven to be crucial in finding an effective way to control their population density and detect the effect of their browsing activity on forest damage (including damage to economically important tree species in the region) [48–51]. The importance of moose browsing and the need to integrate it into management practices were also recently highlighted by Salisbury *et al*. [52]. They demonstrated the role of moose browsing activity in shaping landscape structure and boreal forest composition, with a net impact on climate [52]. Overall, monitoring and predicting moose movements in the landscape are essential for planning both moose and forest management practices, especially in relation to the growing concern about the effects of climate change on animal migration and biodiversity conservation [15, 53–55].

Since both migrant and non-migrant individuals are often present within the same population [56, 57], much attention has been focused on understanding their movement behaviour, often using the GPS tracking system. For example, some researchers have studied the interaction between wolves (predator) and moose (prey) [58, 59]. Others have attempted to detect differences in the moose home-range and behaviour in relation to age and sex [54, 60], season [54, 61], snow quality and depth [56, 62], foraging strategies [63] and habitat deterioration [64, 65].

Most research on moose has focused on a single population living in a restricted area of Scandinavia (but see [47]). Here we present a large amount of samples distributed throughout Sweden. This represents a rare opportunity to study the mobility of a large herbivore such as moose on a large scale. To date, isotopic analyses on moose samples have been conducted for ecological studies focused on diet, using mainly N (δ^15^N) and C (δ^13^C) stable isotopes [66–70]. To our knowledge, this is the first time that Sr and O isotopes are used together to analyse moose large-scale mobility in Sweden. Furthermore, no one has yet attempted to build a complete multi-proxy Sr isoscape of Scandinavia, here developed through a machine-learning model and literature data. In fact, although much attention has been focused on Sr isotope analysis for past mobility studies in Scandinavia (see among others [71–77]), today only baselines of constrained areas (i.e., mainly those characterised by archaeological discoveries) are available [78–80].

The Sr, C, and O isotope analyses on modern moose samples will be useful as an integrative approach to modern ecological studies on this animal. They will complement traditional methods of tracking mobility (e.g., radio-, satellite- and GPS-tracking) as well as genetic analyses [81, 82], providing a new tool for management and conservation practices. The methodology used here can also be applied to areas other than Scandinavia and to animals other than moose, becoming a useful tool for global biodiversity conservation in the current period of environmental change. Moreover, this approach could be transferred to the study of faunal and human mobility in the past, implementing the growing research field of geostatistics applied to archaeological research (see e.g., [83]).

## 2. Isotopes for tracing mobility and diet

Strontium is an alkaline earth element with four naturally occurring isotopes: ^84^Sr (∼0.56%), ^86^Sr (∼9.87%), ^87^Sr (∼7.04%) and ^88^Sr (∼82.53%). All but ^87^Sr are stable, while this latter is radiogenic, forming by the *β*-decay of ^87^Rb with a half-life of 4.88 x 10^10^ years. Strontium becomes incorporated into the local ecosystem through bedrock weathering, being transferred to soil, where it mixes with groundwaters, surface waters and atmospheric depositions. Sr is then transported to the oceans mainly from rivers as dissolved ions in water or through transport of sediments. Bioavailable Sr is taken up by vegetation through the root uptake and by animals through food and drinking water, with limited fractionation; moreover, any eventual isotope effect is corrected after mass bias normalization to an internal stable isotope ratio (i.e. ^88^Sr/^86^Sr or ^86^Sr/^88^Sr) [84–87]. Sr substitutes then for calcium in the hydroxylapatite [Ca_10_(PO_4_)_6_OH_2_] of vertebrate skeletal tissues. Taken together these characteristics make ^87^Sr/^86^Sr a powerful tool to trace geographically biological [88] and environmental materials [85, 89].

Oxygen has three naturally occurring stable isotopes: ^16^O (99.757%), ^17^O (0.038%), ^18^O (0.205%). Because of their higher abundance, the ratio between the heavy isotope ^18^O to the light isotope ^16^O of a particular material (δ^18^O) is commonly determined in geochemistry [90, 91]. The oxygen in mammalian body water comes mainly from drinking water, but also from oxygen structurally bound to organic compounds in food and atmospheric O_2_ inhaled by respiration [92–94]. The δ^18^O (and its fractionation) of these sources is affected by multiple factors (e.g., precipitation, temperature, humidity, continentality, altitude, latitude), which differ from one geographical area to the other [26, 38]. The δ^18^O has thus become key in provenance studies in archaeology, used for example in the study of seasonal vertical transhumance [95] and paleoenvironmental reconstruction [96], often in combination with other isotope systems [97].

Carbon has two stable isotopes, ^12^C (98.93%) and ^13^C (1.07%). ^13^C has a mass 8.36% greater than that of ^12^C, causing the C isotopes to fractionate in chemical and biological processes [91, 98]. The δ^13^C value of plant tissues mainly changes depending on the CO_2_ fixation process used [99,100], allowing the distinction between C_3_ and C_4_ plants [101]. In addition, the δ^13^C value can be influenced by the so called “canopy effect”, which causes a depletion of the δ^13^C of forest plants due to a number of factors including reduced light levels and re-assimilation of ^13^C-depleted CO_2_ [102, 103]. The δ^13^C is widely used in archaeological research both for dietary and paleoenvironmental reconstruction [101, 104], but also for the study of animal management and faunal migration in combination with δ^18^O and ^87^Sr/^86^Sr values [105, 106].

## 3. Materials and methods

### 3.1 Study area

Scandinavia, known as the Scandinavian Peninsula, is a region in Northern Europe characterised by diverse lithologies and a complex geological history, which has shaped this landscape since the Archean. The bedrock is composed of three primary components categorised based on the timing of rock crystallisation, deposition, and crustal growth: 1) Precambrian crystalline rocks forming the Baltic Shield also known as the Fennoscandian Shield, 2) the so-called Caledonides, rocks of the Caledonian orogeny (0.5–0.4 giga-annum; Ga), and 3) Phanerozoic to Neoproterozoic sedimentary rocks [107].

The Precambrian basement, primarily composed of granites, gneisses and greenstone belts forms the core of the Scandinavian Peninsula. It is exposed in large areas of Norway, Sweden and Finland. The oldest rocks, dated between 2500 and 3100 mega-annum (Ma), are found in the northeast portion of the Fennoscandian Shield, in the Kola Peninsula, Karelia and northeastern Finland. Archean rocks of 2600 to 2800 Ma also outcrop in the northernmost part of Sweden. Metasedimentary and metavolcanic rocks, as well as multiple generations of granitoids, hosting important ore deposits, dated between 1750 and 1900 Ma, formed during the Svecofennian orogeny and outcrop mainly in northern and central Sweden as well as in the southwestern part of Finland. Part of the Baltic Shield is also the Transscandinanavian igneous belt (TIB), consisting of largely undeformed granitoids and associated porphyries, emplaced between 1850 and 1650 Ma. This is a ∼ 1400 km long belt running across the Scandinavian Peninsula, from Småland in southern Sweden through Värmland and western Dalarna and continuing under much of the Caledonian nappes up to northern Scandinavia, cropping out in small tectonic windows [108, 109].

The Caledonides were formed during the collisional event between the continents of Baltica and Laurentia around 450–400 Ma ago, and stretch through most of Norway and in the mountainous northwestern part of Sweden. Sedimentary and volcanic rocks were deposited in the Iapetus Ocean, between the late Proterozoic and Silurian periods, 700 to 400 Ma ago, and during the Caledonian orogeny were thrusted eastwards over the Fennoscandian Shield together with slices of the crystalline basement. The Caledonian rocks consist of high-grade metamorphic rocks and (meta)sedimentary rocks also partially intruded by magmatic rocks [110, 111].

Sedimentary rocks from the Phanerozoic, less than 545 Ma, are found on top of the Precambrian shield region. Sandstones, shales and limestones dated between 540 and 420 Ma ago outcrop across extensive areas in southern Sweden, including the islands of Öland and Gotland. Mesozoic and Tertiary sediments (younger than 250 Ma) are found in southernmost Sweden (Skåne) and in Denmark [112].

A relatively small outcrop of Permian magmatic rocks ca, 250 Ma old forms the Oslo Graben, a failed rift system created during the Variscan orogeny [113].

### 3.2 Sample collection and description

**Fig 1:**
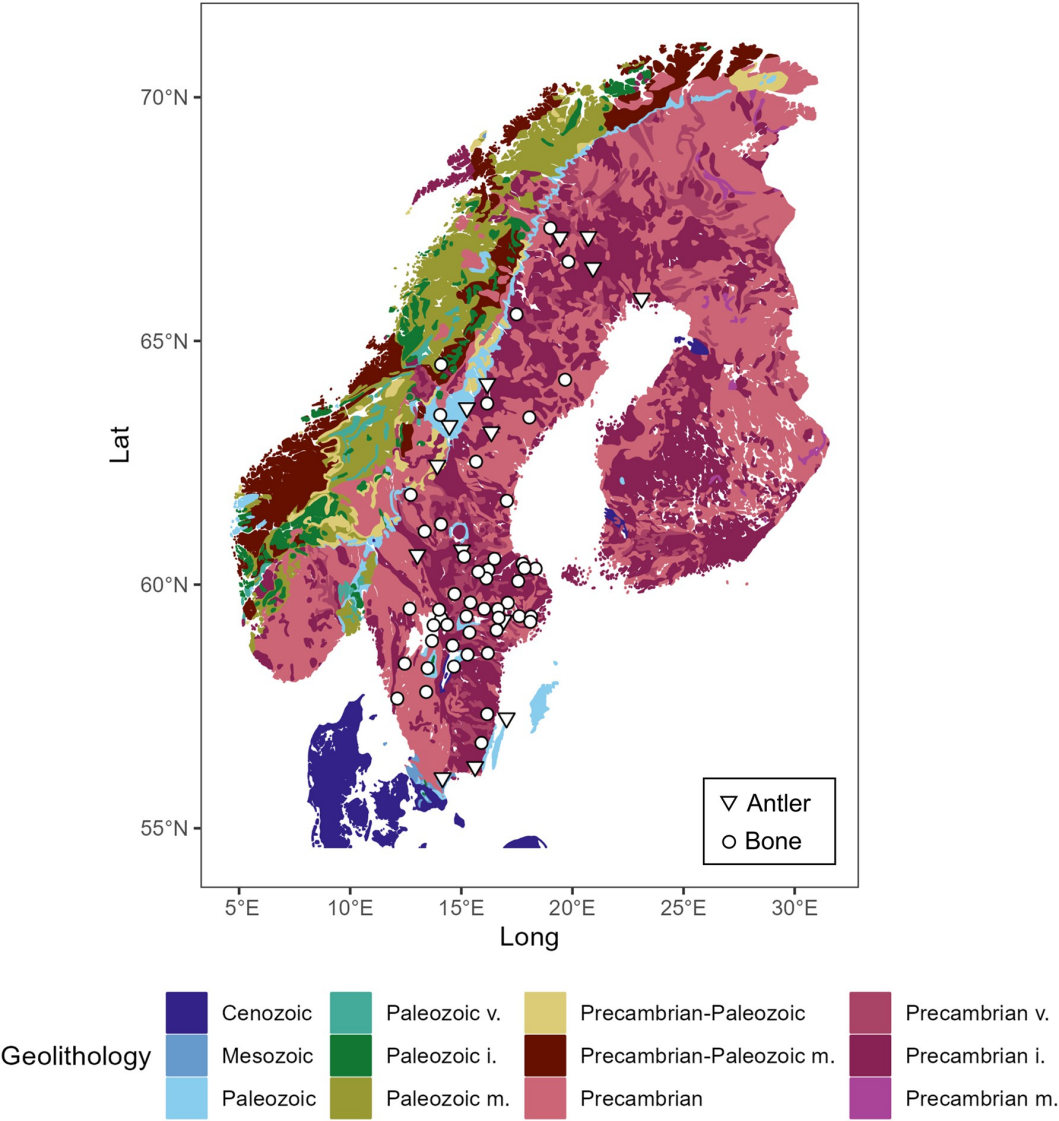
Samples distribution across Sweden. Moose bones (white dots) and antlers (white triangles) are plotted over the geolithological map of Scandinavia. Main stratigraphic units by age are reported; type of lithology is indicated when explicitly reported in the USGS source file: v = volcanic, i = intrusive, m = metamorphic. The map is based on the European geo4_2l shapefile from USGS [114].

Moose bone and antler specimens of adult individuals were sampled in the zoological collection of the Naturhistoriska Riksmuseet in Stockholm, Sweden. No permits were required for the described study, which complied with all relevant regulations. About 50–100 mg powder of bone or antler were drilled manually using a handheld Proxxon drill with diamond studded drill bits. Samples were mostly taken from crania (n = 50) and some from antlers (n = 15), collected covering different bedrock types across Sweden (Fig 1). Antlers were sampled in a cm-size area close to the base (lower part of the antler), representing an estimated period of life less than one month during spring. Overall, n = 29 samples are males, n = 17 females, and n = 19 of unknown sex. Details about the sampled specimens such as sample ID, geographic location, year of death are provided in S1 Table. Bones and antlers have different formation times (i.e., several years and a few months, respectively; see below). For this reason, we have interpreted and discussed the results separately. In addition, we acknowledge that because we are dealing with bone and antler samples from different individuals, our interpretation concerns general aspects of moose ecology, rather than individual-level inferences.

The spatial distribution of the analysed moose samples is reported in Fig 1 and plotted over a geological map of Scandinavia. Latitude and longitude values for each sample are reported in S1 Table.

### 3.3 Isotope analyses

Strontium (^87^Sr/^86^Sr), oxygen (δ^18^O) and carbon (δ^13^C) were measured on a total of 65 bone and antler samples of modern and historical wild-shot *Alces alces* from Sweden. The O and C isotopes were measured on the carbonate portion of the skeletal tissues by using a Gasbench II coupled to a Delta Plus XL IRMS at the University of Tübingen (Tübingen, Germany). Prior to isotope analysis 10 mg of bone powder was pretreated with 2M NaOCl and 0.1 M acetic acid according to the protocol of Koch *et al*. [115] in order to remove organic matter [116]. About 2 mg of pretreated bone powder was reacted for 90 mins at 70°C with phosphoric acid and normalised to a calibrated Laaser marble standard. As quality controls two international carbonate reference materials were measured yielding values in line with certified oxygen and carbon isotope compositions (NBS 18 δ^18^O_VPDB_ = -23.13±0.09‰, δ^13^C_VPDB_ = -4.97±0.04‰, n = 3; NBS 19: δ^18^O_VPDB_ = -2.22±0.12‰ and δ^13^C_VPDB_ = +1.93±0.10‰, n = 3). VPDB values were converted to δ^18^O_VSMOW_. Carbonate δ^18^O values were converted in phosphate (δ^18^O_PO4_) ([117]; all mammals; δ^18^O_c_ = 1.037*δ^18^O_PO4_ + 8.57) and then in δ^18^O of ingested water (δ^18^O_w_) by using the formula for deer of D’Angela and Longinelli [118]. Given the lack of species-specific conversion-equations for moose, the deer equation of [118] represents a close approximation in terms of taxonomy (*Cervidae*) and dietary behaviour (both ruminant herbivores). Carbon isotope values were corrected for anthropogenically induced variation of CO_2_ δ^13^C and reported to a 1994 year-value, by following the workflow of Long *et al*. [119], but using a LOESS model to fit the data of Francey *et al*. [120]. The δ^13^C data reported to a 1800 year-value are also listed in S1 Table. Sr isotopes were measured at the Geochemistry Lab of the Department of Chemical and Geological Sciences (University of Modena and Reggio Emilia; https://www.geochem.unimore.it/). About 5 mg of bioapatite powder for each sample was dissolved in 3M HNO_3_. The Sr separation from the matrix was performed through chromatographic Teflon columns filled with 30 μl of Eichrom Sr spec resin [121, 122]. Once the columns were cleaned with MilliQ, the dissolved samples were loaded and the cations (not Sr) were desorbed by percolating 3M HNO_3_, to eliminate matrix ions. Strontium was then eluted using MilliQ. The Sr samples were diluted with 4% HNO_3_ at 50 ppb and analysed with a Neptune MC-ICPMS at Centro Interdipartimentale Grandi Strumenti of the University of Modena and Reggio Emilia. ^82^Kr, ^83^Kr, ^84^Sr, ^85^Rb, ^86^Sr, ^87^Sr and ^88^Sr m/z were collected with 10^11^ and 10^12^ (for ^82^Kr, ^83^Kr and ^84^Sr) Ω resistors. Background subtraction and Rb correction was performed with routine methods (see [123]); similarly, mass bias normalisation used an exponential law and an ^88^Sr/^86^Sr ratio of 8.375209 [124]. Samples’ ^87^Sr/^86^Sr ratios were reported to an accepted NIST-SRM 987 value of 0.710248. Repeated analyses of the NIST-SRM 987 yielded an average ^87^Sr/^86^Sr ratio of 0.710228 ± 0.000018 (2 SD, n = 20).

### 3.4 Geostatistical framework

Several studies have been conducted to determine which materials are most suitable for establishing the local Sr baseline and building the isoscapes [125]. Today there is general agreement on the preferential use of archaeological microfauna, snails, modern plant and water samples [36, 37, 126, 127]. Other natural materials useful for provenance studies include soil leachates [128] as well as modern and archaeological faunal tooth enamel and bones, provided they are local to the area under study and free of any contamination or diagenetic modification [36, 129].

Given these assumptions, the first step in building the Sr isoscape was to collect bibliographical data of natural samples’ ^87^Sr/^86^Sr, including vegetation, waters, soil leachates, snails, animal bones and teeth (both modern and archaeological) from Sweden and surrounding countries (i.e., Norway, Finland and Denmark; see S2 Table). Only local fauna (as described in the respective paper) was selected, including snails, microfauna, animals with a small home-range, and domestic animals. Among the natural samples, we preferably selected plants and waters as the more representative of the local bioavailable Sr pool. Soil leachates were added only if no other type of sample was available for a given site. Moreover, since as pointed out earlier the ^87^Sr/^86^Sr values are only available for limited areas of Scandinavia, we added the ^87^Sr/^86^Sr values of modern soil leachates from GEMAS project [128] to our database to cover as many territories as possible. All the further data analyses were carried out in R (version 4.0.5).

To build the Sr isoscape map of Scandinavia we used Random Forest regression (RF) with multiple predictors (*randomForest* package; [130]), following the method of Bataille *et al*. [40]. The Random Forest is a supervised tree-based machine learning algorithm that uses a labelled database including environmental and geological information to predict the isotope ratios in areas with similar features [39, 40, 131]. Seven external variables, obtained from global raster maps (see [40]), were selected by *VSURF* [132] based on their importance in predicting the ^87^Sr/^86^Sr ratio. After the map outline, a 10-fold cross-validation was performed to estimate the power of the prediction, evaluated as RMSE (Root mean square error). To generate a spatial-uncertainty map, we employed a quantile RF regression (*ranger* package; [133]), then halving the RF q_0.84_ ‐ q_0.16_ difference (i.e., lower and upper limits of a ∼68% interval; [134]). The Sr isoscape and the error map can be found as S1 and S2 Files, respectively. To test the Sr natural variability vs. the RF error, we gathered all the data with the same coordinates from S2 Table and calculated the ^87^Sr/^86^Sr standard deviations (SD) from each site. As depicted in S1 Fig, both the observed SDs (S1A Fig; R^2^ = 0.46, p < 0.01) and the calculated RF error from the error map (S1B Fig; R^2^ = 0.74, p < 0.01) are correlated with the mean ^87^Sr/^86^Sr of the site. This suggests that both errors and isotope variability are higher in high-radiogenic areas, as expected (see e.g., [39, 131]). The observed SD among the samples and the calculated RF error from the error map are also correlated with each other (S1C Fig; R^2^ = 0.36, p < 0.01), with an average difference of 0.0024 ± 0.0043. The RF error tends to underestimate the observed local isotopic variability at sites with elevated ^87^Sr/^86^Sr ratios (S1C Fig).

The oxygen isoscape (annual average value) was downloaded from *waterisotopes*.*org* and cropped for the area of interest. This represents the mean modelled climatological prediction based on annual precipitations [20]. A conservative 1‰ constant spatial uncertainty was arbitrarily associated with each pixel of the oxygen isoscape. This error is 10-times larger than the median error associated with the model (∼0.1‰) and compatible with the spatial uncertainty used in other works (e.g., [135–137]).

To assess the ‘local vs non-local’ isotope signature, we checked the difference between the isotope ratio of the samples themselves and the isoscapes at their position (Δ^87^Sr/^86^Sr_sample-isoscape_ and Δ^18^O_sample-isoscape_). To do so, we extracted and averaged pixel values from the maps at sample location, with a buffer radius of 10 km. The buffer value to be used was chosen, testing different radius lengths (from 1 km to 250 km) and selecting the resulting linear model (ordinary least square of sample vs. isoscape isotope ratios) with the highest coefficient of determination (see S3 Table) and compatible with expected moose home ranges from ecological data (see Discussion). Samples within 1 standard-deviation (1σ thereafter) of the Δ^87^Sr/^86^Sr_sample-isoscape_ and Δ^18^O_sample-isoscape_ distributions were thus considered as compatible with the local area (10 km radius buffer), also accounting for the isoscape(s) spatial uncertainty (see Results). Both the isoscapes were then calibrated using a linear fit with the moose samples within 1σ (i.e., those likely ‘local’); values outside 1σ-variability of either Δ^87^Sr/^86^Sr_sample-isoscape_ or Δ^18^O_sample-isoscape_ were thus removed from the calibration.

Samples outside 1σ-variability of the isoscape Δs, thus likely different from the local-buffer area, were compared with the isoscapes using a Bayesian probabilistic approach and the ‘*assignR*’ package in R [138]. The prior probability assumes that all grid cells are equally likely locations of origin of these samples. The posterior probability of origin is computed at each grid cell, returning a raster object which contains one probability density surface per sample with its likely provenance. In the calculations, raster maps with prediction errors of the modelled isoscapes are also included. The Sr and O obtained posterior probabilities were joint to obtain a final combined dual-estimation.

To test the effect of forest cover on δ^13^C of moose samples, pixels of a Sweden forest cover map (from [139]; time span 0–125 years BP) were extracted with a 10-km buffer radius at the (moose) sample locations. The forest cover maps of [139] are based on remote-sensing data, calibrated through (fossil) pollen records and represent a full range of pixel values from 0 to ∼90% canopy closure. Then, ordinary least square models of sample δ^13^C tissues vs. %-forest cover were calculated; ‘non-local’ moose based on Sr-O were excluded.

The R code is available online on Zenodo: https://zenodo.org/records/10418660.

## 4. Results

**Fig 2:**
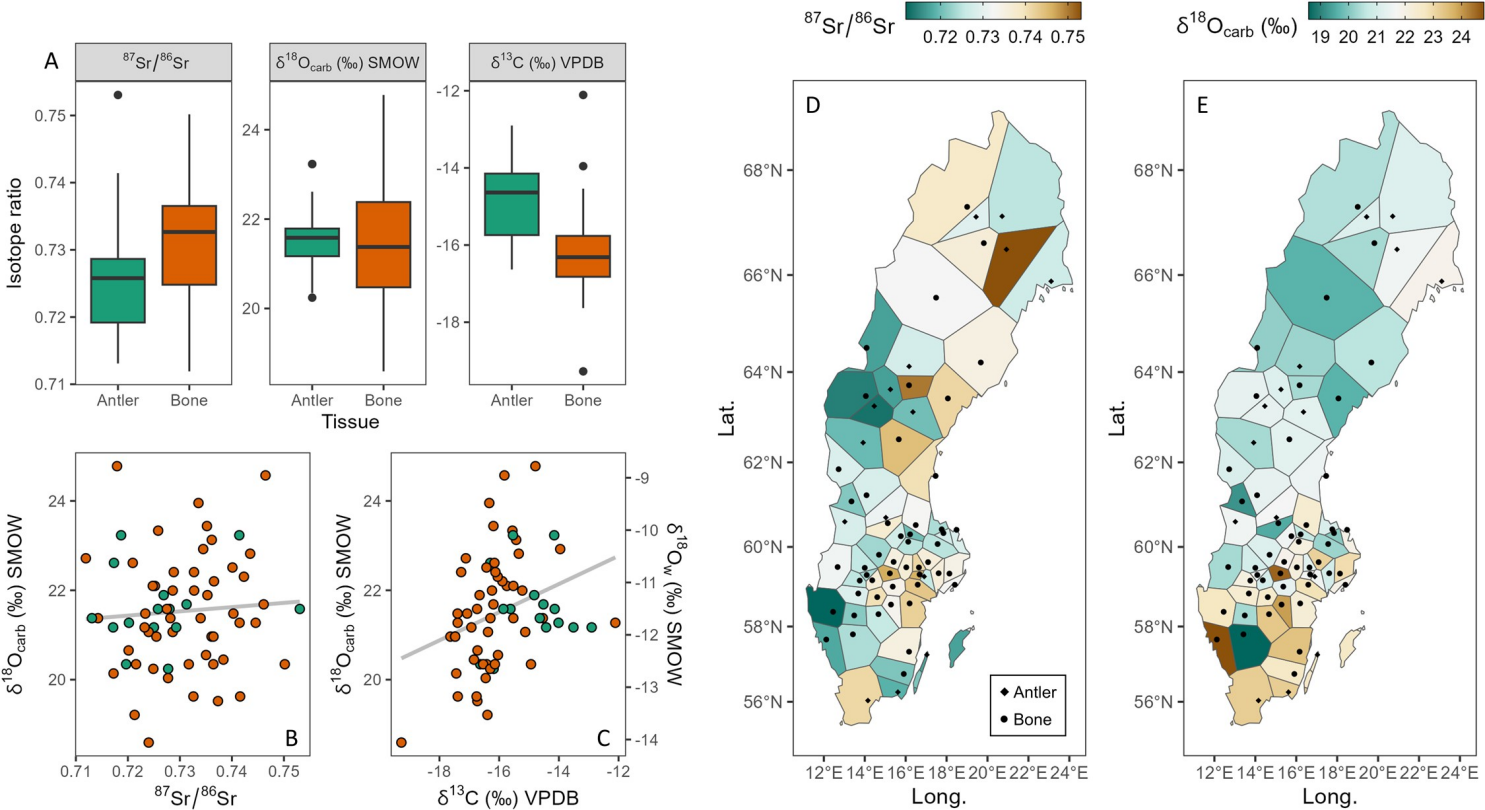
Strontium, oxygen and carbon isotope data of moose antlers and bones. A) Boxplot of strontium, oxygen and carbon isotope data of antlers (green; n = 15) and bones (orange; n = 50). B) δ^18^O values of antlers and bones versus their respective ^87^Sr/^86^Sr and C) δ^13^C values; color coding as in (A); grey lines are linear regressions through the data. D) Voronoi diagram of moose’s Sr isotope values plotted over the map of Sweden. E) Voronoi diagram of moose’s oxygen isotope values plotted over the map of Sweden.

The complete list of isotopic results, along with all the information about the samples, are available in S1 Table. Strontium, oxygen and carbon data are shown in Fig 2(A)–2(E). Since bone and antler represent different time averages (i.e., several years and a few months, respectively) [69, 140] we decided to present bone and antler data separately.

Strontium isotopes (^87^Sr/^86^Sr) of moose antlers range between 0.71309 and 0.75305 with a mean value of 0.72631 (± 0.01022, 1 SD, n = 15). Bones yielded also a similarly wide range with ratios between 0.71192 and 0.75017 but the mean value is more radiogenic and equals 0.73133 (± 0.00887, 1 SD, n = 50). Such radiogenic values clearly reflect the geology of Scandinavia, dominated by old rocks.

**Fig 3:**
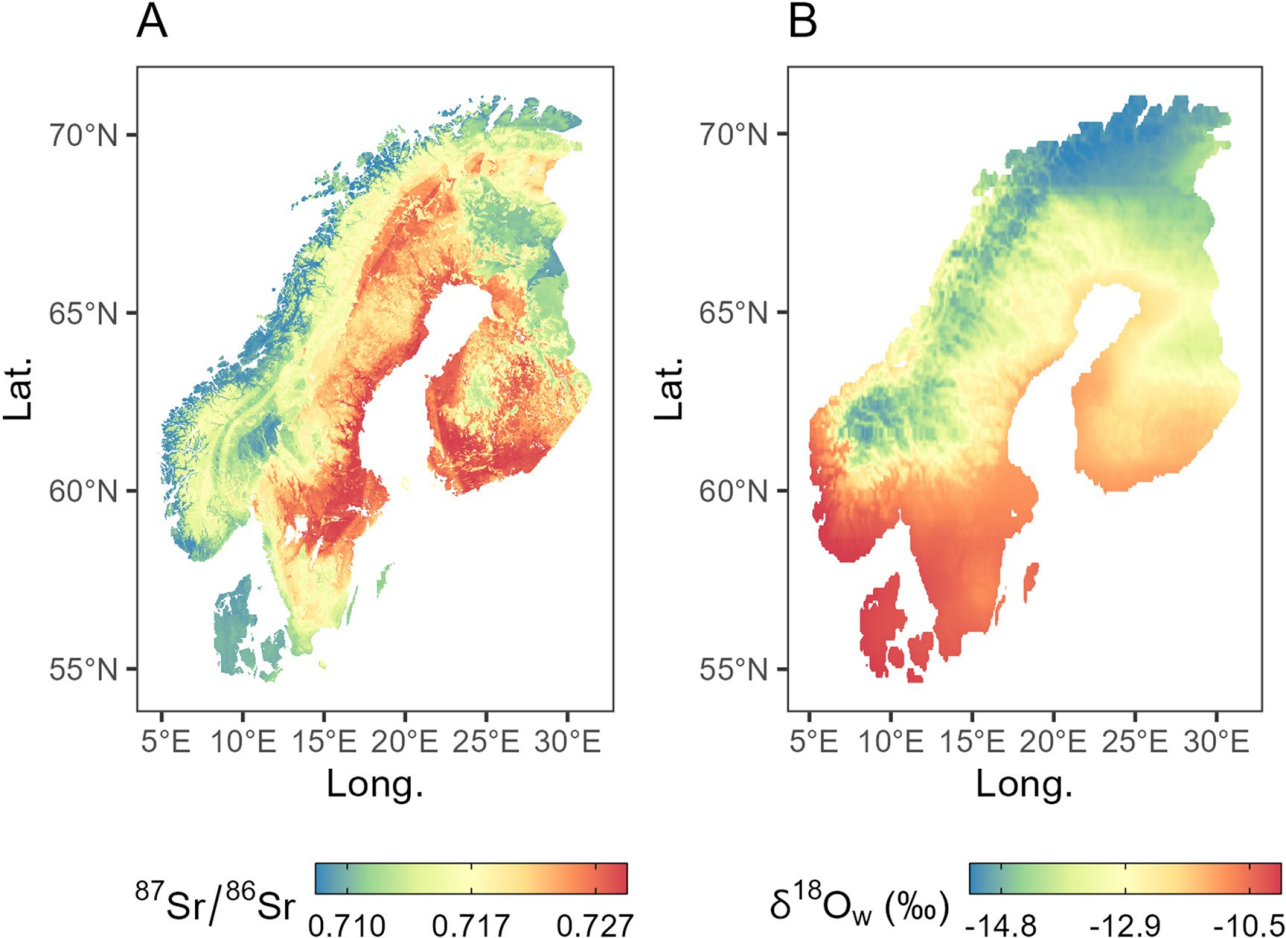
Isoscapes. A) ^87^Sr/^86^Sr isoscape built through Random Forest with n = 7 external predictors; B) δ^18^O isoscape (mean annual precipitation values) downloaded from *waterisotopes*.*org*. Maps are colored through a quantile scale (q_0.1_, median, q_0.9_); min and max values for the ^87^Sr/^86^Sr isoscape (A) are: 0.705 and 0.748, respectively; while for the δ^18^O isoscape (B) are -17.3 and -7.1‰.

Oxygen isotopes (δ^18^O_SMOW_) of the carbonate moiety structurally bound in the bioapatite of moose antlers yielded a mean value of +21.6‰ (± 0.9, 1 SD, n = 15), ranging between +20.2 and +23.2‰. Similarly, bones yielded a mean δ^18^O value of +21.5‰ (± 1.3, 1 SD, n = 50), ranging between +18.6 and +24.8‰. Once converted in water values, oxygen isotopes agree with values observed in Scandinavia (see *waterisotopes*.*org* and Fig 3B).

Carbon isotopes (δ^13^C_VPDB_ corrected for CO_2_, see Materials and Methods) of the carbonate portion of bioapatite of moose antlers vary between -16.6 and -12.9‰ with a mean value of -14.9‰ (± 1.1, 1 SD, n = 15). Bones yielded a wider range between -19.3 and -12.1‰ with a mean of -16.2‰ (± 1.1, 1 SD, n = 50). These values are typical of C_3_ plants feeders, in agreement with C_3_ and C_4_ plants distribution in Europe [21, 100, 141]. No statistically significant correlation was found between δ^13^C values of moose tissues and forest cover (bone R^2^ = 0.001, *p* = 0.801; antler R^2^ = 0.03, *p* = 0.52; combined R^2^ = 0.02, *p* = 0.22).

We tested the correlation between oxygen and strontium isotopes (Fig 2B; R^2^ = 0.005, *p* = 0.570), and the correlation between oxygen and carbon isotopes (Fig 2C; R^2^ = 0.098, *p* = 0.011). In both cases there is no significant linear trend.

The Sr isoscape (Fig 3A) obtained through RF with 7 external predictors (*r*.*srsrq3*, *r*.*fert*, *r*.*elevation*, *r*.*ssa*, *r*.*cec*, *r*.*ssaw* and *r*.*mat*, see [40]) yielded RMSE = 0.0055 and R^2^ = 0.65. For additional details about the isoscape see S2 Fig. The modelled isoscape varies between 0.70519 to 0.74820, with a median of 0.71714. Bataille *et al*. [142]’s model product *r*.*srsrq3* is the more dominant predictor, indicating that the bedrock geology and rock age impacted the modelled Sr isotope values. The importance of *r*.*fert* (fertilisation rate) in the prediction is likely due to the fact that GEMAS soils (used in the interpolation) are mainly agricultural soils and thus potentially affected by the use of N- and P-based fertilisers. Sea salt deposition (*r*.*ssaw* and *r*.*ssa*) also strongly contributed to the Sr isotope variability observed in the isoscape (see e.g., the relatively low Sr isotope ratios along the Norway coastline). Locally, elevation (*r*.*elevation*) seems to drive the isoscape ^87^Sr/^86^Sr, with low isotope values at mid-low-elevations (∼200 m; see S2 Fig). This is possibly due to the exposure by tectonic and subsequent erosion of deeper portions of the crust with different lithologies (e.g., see gradient from Norway coast to the Caledonides mountain range).

The oxygen isoscape (Fig 3B) varies between -17.3 to -7.1‰, with a median of -12.9‰. As expected, a latitudinal trend is observed, with the most positive values in the south and the most negative values in the north. This trend reflects the typical distribution of oxygen isotopes in precipitation due to the progressive condensation of the vapour during transport to higher latitudes with lower temperatures. Oxygen isotopes tend to be depleted in ^18^O as elevation increases and this can be seen in profiles from sea level to the mountainous areas of Sweden and Norway.

**Fig 4:**
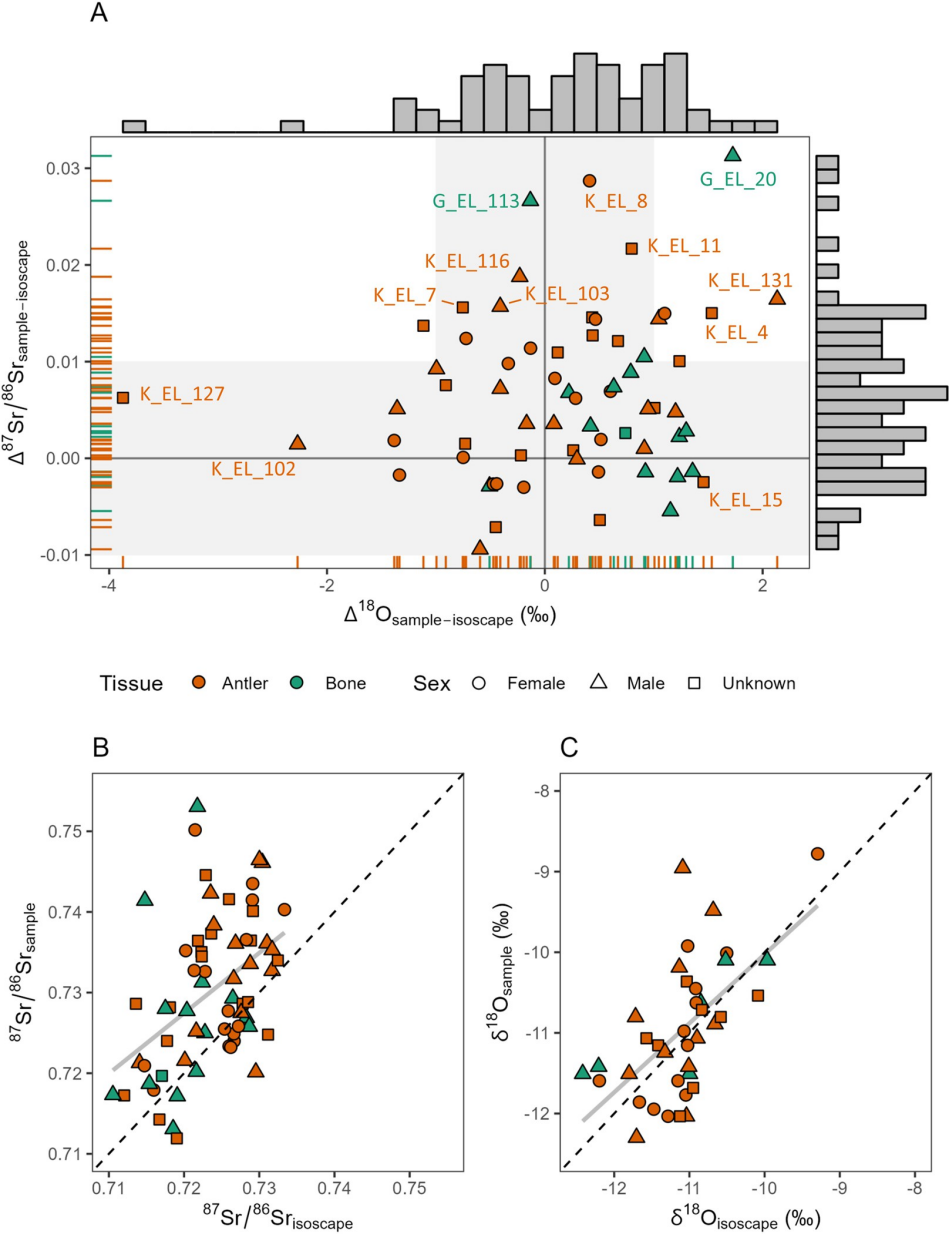
Data comparison between sample and isoscape. A) Bone (n = 50) symbols are orange; while antler (n = 15) symbols are green; males (n = 29) are triangles; females (n = 17) are circles; individuals with unknown sex (n = 19) are squares. One antler is reported as ‘sex unknown’ being labelled as ‘hermaphrodite’ in the museum ID card (see S1 Table). 1σ-outliers are labelled; gray bars on the sides are histograms of univariate sample distribution for Δ^87^Sr/^86^Sr_sample-isoscape_ and Δ^18^O_sample-isoscape_; rug bars are also sample distributions but classified by sample type (bone and antler). Light grey areas are approximate median spatial uncertainties of the isoscapes (∼0.01 for Sr and 1‰ for oxygen) depicted below and above Δ = 0. B) The linear fit (1σ-outliers included) between the ^87^Sr/^86^Sr_sample_ and the ^87^Sr/^86^Sr_isoscape_ shows an R^2^ of 0.20, *p* < 0.01; C) The linear fit (1σ-outliers included) between the δ^18^O_sample_ (water) and the δ^18^O_isoscape_ shows an R^2^ of 0.30, *p* < 0.01.

We calculated the difference between the isotope ratio of the samples and the isoscapes at their position (Fig 4A). The Δ^87^Sr/^86^Sr_sample-isoscape_ yielded a median value of 0.0052, ranging between -0.0094 to 0.0313. The Δ^18^O_sample-isoscape_ yielded a median value of 0.3, ranging between -3.9 to 2.1. No spatial trend is evident plotting the delta over the Scandinavia map (S3 Fig). Overall, this suggests that the Sr isoscape slightly underpredicts the moose Sr isotope biological values. This could be related to several factors such as: 1) the GEMAS dataset is composed by soil leachates, which may show ^87^Sr/^86^Sr shifted to the local carbonate end-member, i.e., less radiogenic values; 2) the isoscape data density is higher in some specific areas (e.g., archaeological sites), and not evenly distributed throughout the region; 3) intrinsic limits of the modelling method itself (see S2 Fig); 4) moose diet is on average more radiogenic than the expected local bioavailable Sr (e.g. ingestion of silica dust; reliance on plants with deep rooting depth); 5) changes in the bioavailable ^87^Sr/^86^Sr across time due to e.g. the eventual loss of forested areas [143]. On the other hand, the oxygen isotope values of the isoscape seem to better predict the sample oxygen isotope values.

We tried to identify any difference in the Δ^87^Sr/^86^Sr_sample-isoscape_ and Δ^18^O_sample-isoscape_ between sexes. We found *p* = 0.89 and 0.04 (non-parametric Mann-Whitney U test), respectively (S4 Fig). This means that the sexual difference in the Δ^87^Sr/^86^Sr_sample-isoscape_ is not statistically significant, while the Δ^18^O_sample-isoscape_ sexual difference is significant at *p* = 0.05. Considering bone samples only, the difference is not significant both for the Δ^87^Sr/^86^Sr_sample-isoscape_ and the Δ^18^O_sample-isoscape_ values (*p* = 0.63 and *p* = 0.77, respectively), suggesting that the observed significant Δ^18^O_sample-isoscape_ sexual difference was driven by the presence of antlers in the dataset. Overall, this indicates that the sex is not a driving factor in the Δ_sample-isoscape_ values. We indeed found a statistically significant difference in Δ^18^O_sample-isoscape_ between antler and bone tissues at *p* = 0.05 (non-parametric Mann-Whitney U test, *p* = 0.001 all sample considered; *p* = 0.03 male only), with median Δ^18^O_sample-isoscape_ of antlers higher than bones (∼1‰). This likely reflects the period of antler growth, namely during warm seasons (spring-summer; [144]), and thus registering relatively higher δ^18^O values of ingested water.

Since most of the Δ^87^Sr/^86^Sr values are positive (Fig 4A) and given the good correlation between the samples and the isoscapes (Fig 4B and 4C), we choose to linearly calibrate both the isoscapes using the samples within ± 1σ. After removing the 1σ-outliers, the linear fit between the ^87^Sr/^86^Sr_sample_ and the ^87^Sr/^86^Sr_isoscape_ shows an R^2^ of 0.35 (*p* < 0.01; intercept = 0.15 ± 0.10; slope = 0.80 ± 0.15), while the linear fit between the δ^18^O_sample_ and the δ^18^O_isoscape_ shows an R^2^ of 0.51 (*p* < 0.01; intercept = -3.4 ± 1.1; slope = 0.70 ± 0.09). The calibrated isoscapes are on average more positive/higher (0.0055 for Sr and 0.50 for oxygen) than the uncalibrated ones (see S5 Fig). Due to the large spatial uncertainties associated with both isoscapes (see Figs 4 and S6), we considered individuals within the 1σ-variability as likely compatible with the local isotope signature, in a buffer-radius of 10 km.

**Fig 5:**
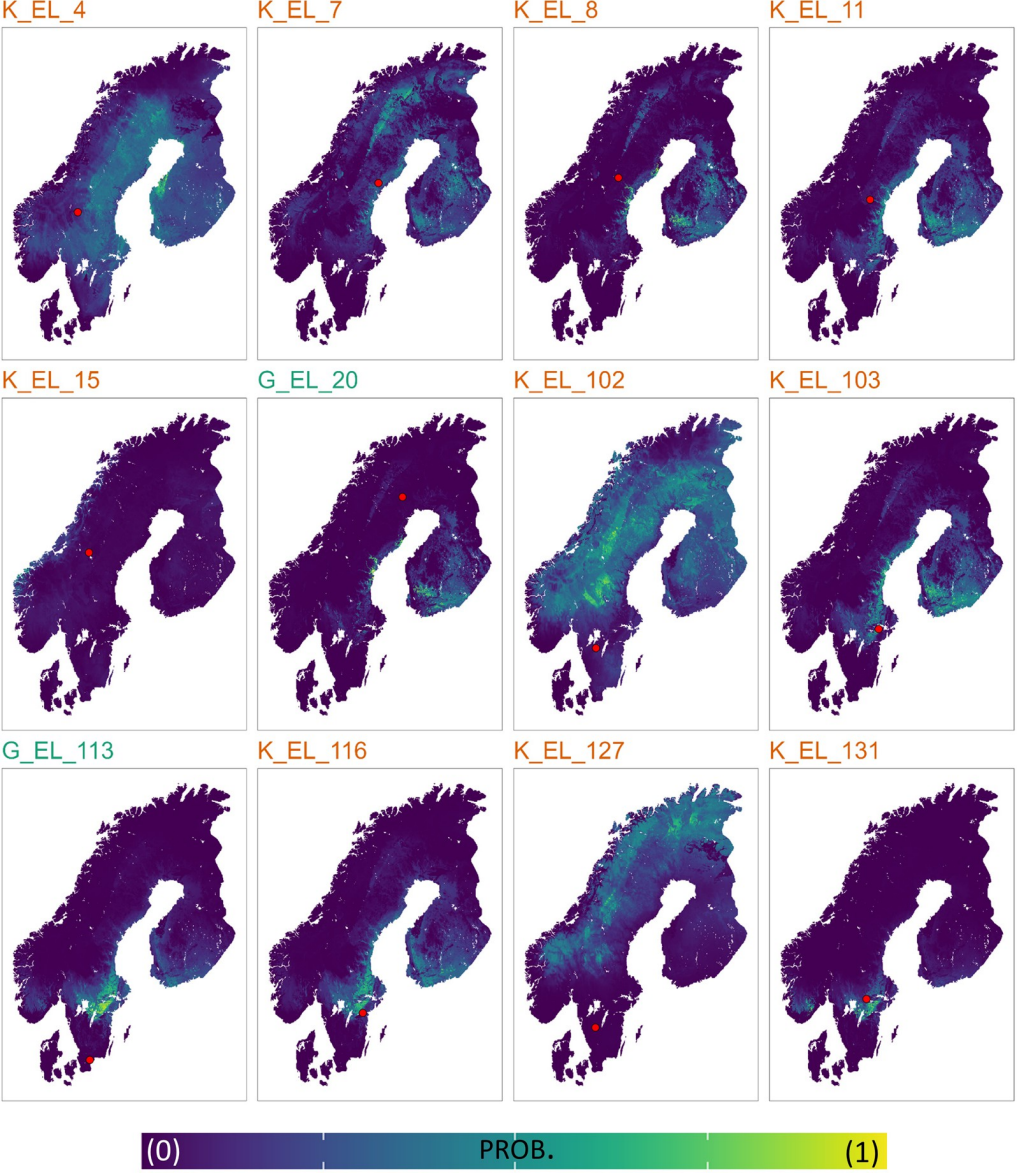
Moose provenance probabilities estimated through a Bayesian approach. Sr and O calibrated isoscapes were combined to predict the place of origin of the 1σ-outliers (see the Materials and Methods section). Red dots are the places of death of the individuals. Probability estimates are scaled between 0 (low probability, purple) and 1 (high probability, yellow). Sample’s names are reported in green for antlers and orange for bones.

**Fig 6:**
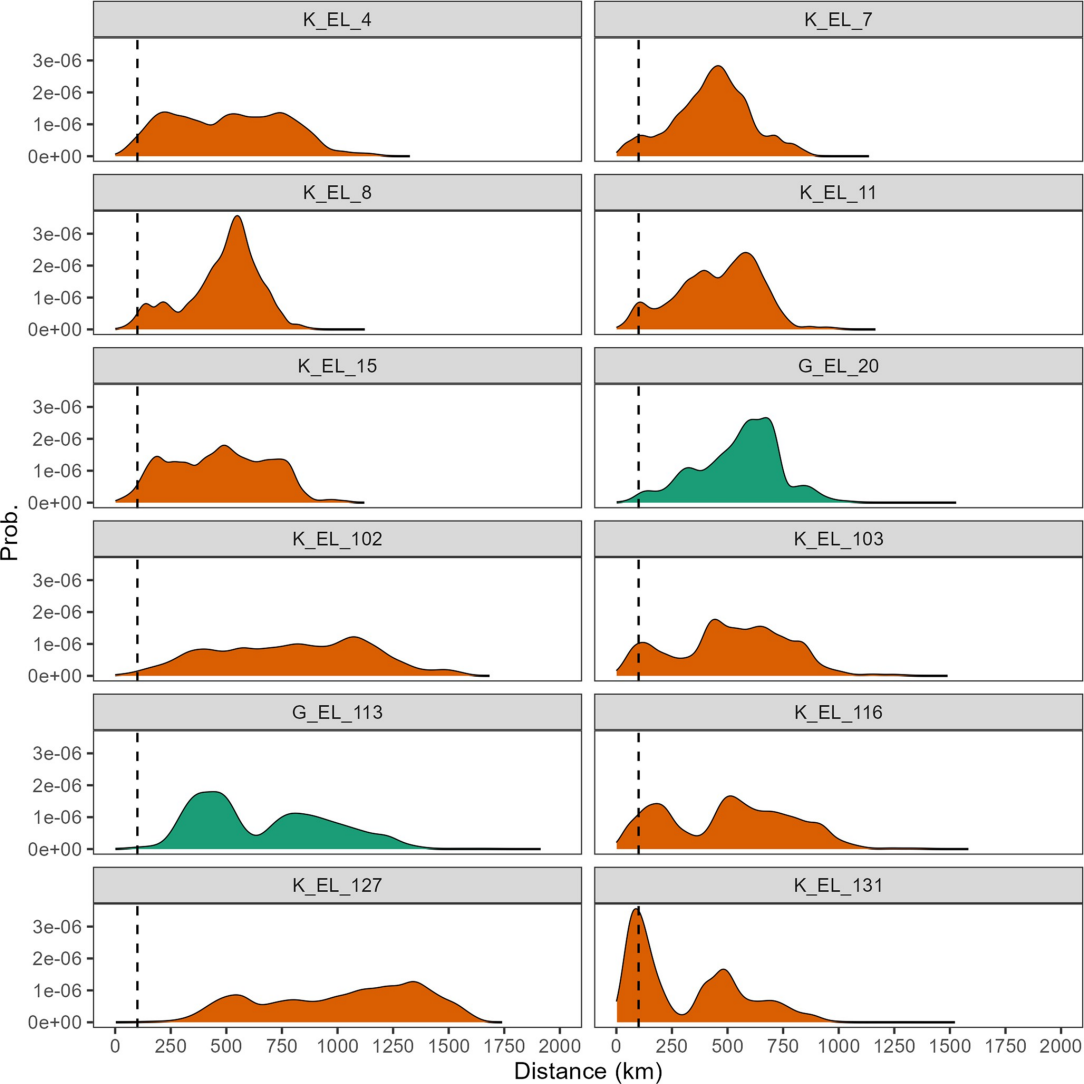
Probability distance of origin. Probability density plots representing the likely distance of movements determined for 1σ-outliers (see the Materials and Methods section) using *assignR* on the calibrated isoscapes. Bones are orange; antlers are green. Dashed line is an arbitrary cut-off value of 100 km (see Discussion).

We used the calibrated isoscapes to test the provenance of the 1σ-outliers and their probability distance of origin (Figs 5 and 6). Although in some cases the probability distributions display the highest values at more than 250 km (up to 1000+ km), the most likely distance travelled is perhaps less than 100 km, i.e., compatible areas closest to the place of death, where increases in probability distributions are already evident (see Discussion below). The only samples for which a higher degree of mobility can be assumed confidently are G_EL_113 (antler) and K_EL_127 (bone). In fact, their probability distributions begin to increase at 250 km of distance.

## 5. Discussion

### 5.1 Strontium and oxygen isotopes: Moose mobility

In the current period of environmental change, the study of faunal mobility across the landscape has become increasingly important. Quantifying and predicting the spatial distribution of species are necessary to enable the conservation of biodiversity and ecosystems [8, 10]. Geochemistry is proving to be a key element in this regard, allowing to track animal movement across the territory. More and more isotopic landscapes have been built in recent years, both for archaeological and ecological applications [131, 145–149].

The newly built Sr isoscape of Scandinavia presented here fits in this research framework, with the goal of tracking moose movements across the landscape. The bedrock age has been shown to be key in controlling the bioavailable ^87^Sr/^86^Sr across Scandinavia. The most radiogenic Sr isotope compositions correspond to areas where Precambrian rocks, particularly those of the Svecofennian orogeny, outcrop that is in most of northern and central Sweden and in southwestern Finland. The Sr isotope composition gradually becomes less radiogenic towards the west, across the Caledonides and down to the Norwegian coast line. The rocks of the Caledonides are clearly not only younger, but also contain ophiolitic sequences that generally have more depleted isotopic compositions. Along the coast of Norway, a key variable is the salt deposition through sea spray effects. Salts here are continuously replenished by oceanic waters. Interestingly, sea salt contribution is none in the Baltic Sea coastal regions most likely due to the competing and major contribution of high Sr isotope signatures from the erosion of the outcropping Precambrian rocks.

It is well known that moose display a wide range of movements (migration, nomadism, dispersal, home range, sedentary) [47, 54, 150]. Variations in movements are visible at the population level (i.e., some individuals migrate while others do not), but also during the lifetime of a single individual (e.g., in relation to age). Many reasons were identified by scholars for moose movements in Scandinavia, including seasonality, forage availability and nutritional needs, climatic factors, snow depth and quality, sex differences (e.g., reproductive status, presence of calves), habitat deterioration, human presence in the landscape [46, 47, 56, 59, 60, 63, 65, 151]. So far, the usefulness of genetic analysis as an adjunct to ecological research has been highlighted [81, 82]. Geochemical analyses can add new and complementary information on moose mobility behaviour, both at a seasonal (antlers) and at annual level (bones).

We first note that the isotopic values of bones (mainly cranial bone) represent a mean of several years of life of the individual. On the other hand, the antlers provide an isotopic signal corresponding to their growing season (between spring and summer) [69, 152, 153]. In the present study, antlers were sampled close to the base, thus likely reflecting ∼1 month of life in spring. Sr and O data of most of the moose samples (within 1σ-interval of Δ^87^Sr/^86^Sr_sample-isoscape_ and Δ^18^O_sample-isoscape_) are likely compatible with the isotopic signature of the place of death, thus indicating a narrow home range. The 1σ-outliers whose provenance through the calibrated Sr-O isoscapes has been estimated include ten bones and two antlers (see Figs 5 and 6). Although in some cases the probability peaks begin to increase around 250 km away from the place of death and some samples display the highest peaks around 500 km distance, in the light of the ecological data on this animal (see below) it is reasonable to assume that the likely distance travelled is represented by the probability peaks between 0 and 100 km (see dashed line in Fig 6). However, it is possible that at least some individuals travelled longer distances (i.e., above 100 km; see Fig 6).

Available information on moose annual home range in Scandinavia shows that both migratory and nonmigratory individuals can travel mean maximum distances of approximately 5 to 28 km (diagonal of hypothetical square-shaped home ranges 12.6 and 410 km^2^) [50, 58–61, 154, 155]. Similar values have also been documented in Canada and the United States, where the maximum distance travelled, obtained from annual home ranges of 20 to 300 km^2^, spans from 6.3 to 24.4 km [156–161]. On the other hand, spring and summer home ranges of both migratory and nonmigratory moose in Scandinavia are reported to be from less than 1 to about 30 km^2^, corresponding to a mean maximum distance travelled of 1.2 to 7.7 km [60, 61, 63, 162]. Higher distances have been reported for Canada and the United States. Here moose occupy seasonal ranges above 20 km^2^ and up to 600 km^2^, corresponding to maximum distances of 7 to 17.3 km [57, 156, 159].

During spring and fall migrations instead, moose in Sweden have been documented to travel a minimum of 4.4 up to 217 km [47, 163]. Migratory movements of 100 up to 500 km have been documented in Alaska, Siberia and Altai mountains [42, 164]. Long-distance dispersal of up to 1,500 km has been documented in the central United States [165]. Moose migrations over long distances have been confirmed by genetic analysis in both Scandinavia and continental Europe, showing a statistically significant gene flow at 300–400 km and 400–500 km, respectively [82]. In this sense, our most interesting samples are G_EL_113 (antler) and K_EL_127 (bone). Their probability distance of origin begins to increase from 250 km, reflecting larger-scale movements both seasonally (spring/summer) and annually. We emphasize here that G_EL_113 (non-local) provenance mainly relies on a remarkably high radiogenic Sr isotope value of the sample. As reported in the geostatistical framework section, the Sr Scandinavian isoscape shows the highest prediction uncertainties in high radiogenic areas and, thus, we interpret the provenance of this sample with caution. Overall, more work is needed to accurately link the ^87^Sr/^86^Sr ratio of moose with Scandinavian bioavailable Sr baselines. This, in turn, indicates a need for more bioavailable Sr samples from radiogenic areas, as well as better statistical methods to estimate uncertainty in such highly variable regions.

Migratory patterns in moose can differ between male and female individuals. In our dataset, differences between the sexes are negligible. Although female moose are less represented, it is noteworthy that 6 out of 12 of the 1σ-outliers are males. The others are 1 female and 5 of unknown sex. This likely higher rate of movement of males than females agrees with the ecological data. Indeed, many scholars have found sexual differences in home range size and migration distance, both in relation to age and nutritional demand, with males travelling farther than females [47, 54, 60, 155].

**Fig 7:**
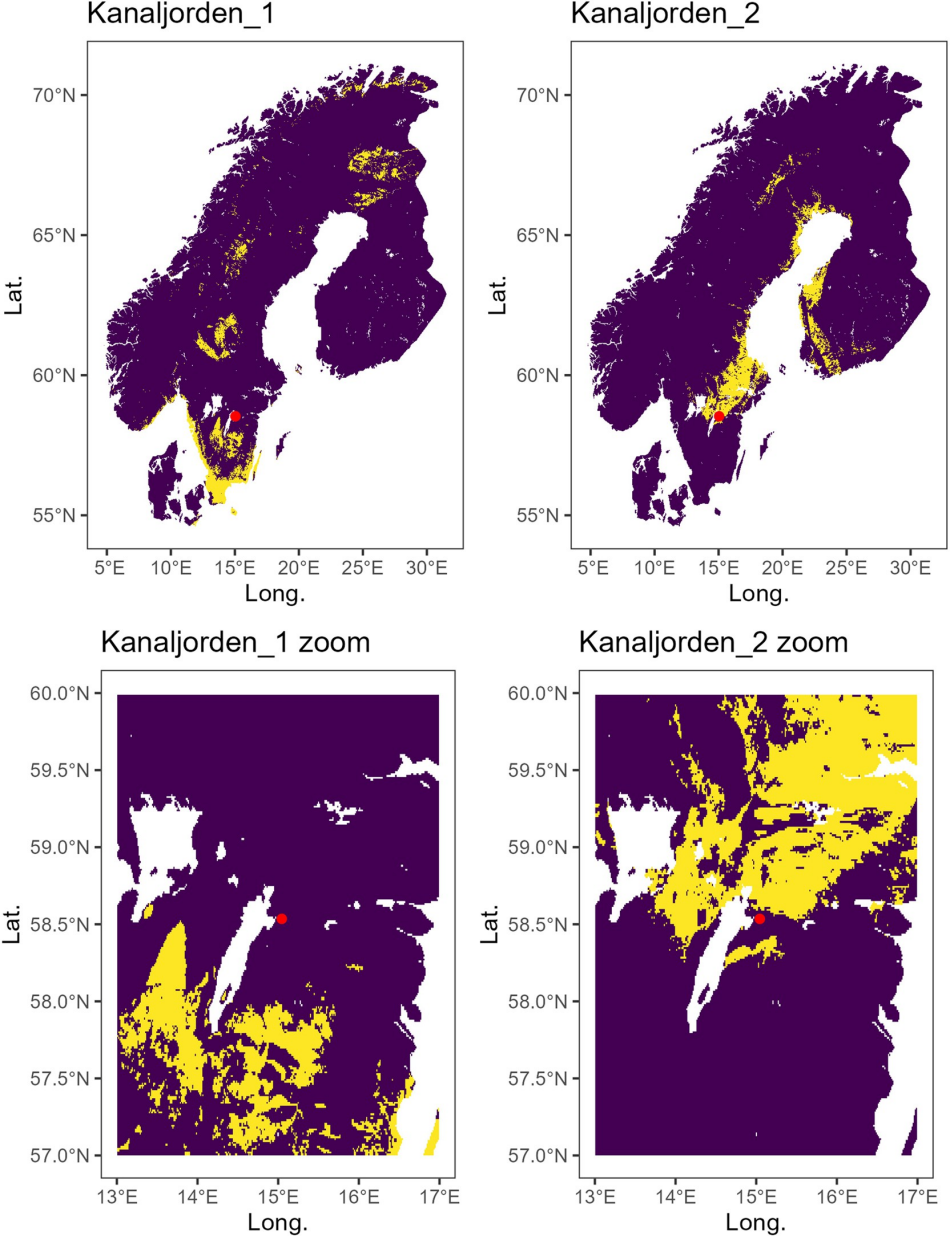
Top 5% probability of origin of two archaeological moose samples based on their Sr isotope ratio. Sample Kanaljorden_1 is a tooth pendant (enamel ^87^Sr/^86^Sr = 0.72086) while Kanaljorden_2 is an incisor (enamel ^87^Sr/^86^Sr = 0.73489). Data are from Eriksson *et al*. [166]. The red dot is the site of Kanaljorden. Yellow represents highly probable areas of origin (top 5%, see [138]), while purple represents low probable areas of origin.

Due to the remarkable implications of this approach for paleoecological and archaeological studies in Scandinavia, we tested our calibrated Sr isoscape on Swedish archaeological materials. We assessed the provenance of two moose samples from the Mesolithic Kanaljorden site in Motala previously published by Eriksson and colleagues [166]. These samples consist of a tooth pendant (^87^Sr/^86^Sr = 0.72086) and an incisor (^87^Sr/^86^Sr = 0.73489) defined as local and non-local to the site, respectively. The main issue with this assessment is that it was made using a local Sr baseline defined primarily by soil leachates. The leachable fraction of soils can be indeed less radiogenic (i.e., shifted to the local carbonate pool) than the local bioavailable ^87^Sr/^86^Sr [167]. For this reason, we propose that the ^87^Sr/^86^Sr local baseline assessed by Eriksson *et al*. [166] is possibly underestimated. Using our calibrated isoscape the provenance of the two moose samples changes. The more radiogenic incisor (Kanaljorden_2) appears to be local to the site, while the less radiogenic tooth pendant (Kanaljorden_1) is probably of non-local origin and compatible with an area about 100 km south of the site (Fig 7).

### 5.2 Carbon isotopes: Diet

Other key aspects of moose ecology, feeding habits and habitat selection, can be revealed by carbon stable isotopes [67, 69]. The mean δ^13^C_VPDB_ values (corrected for the ‘Suess effect’) of the carbonate portion of bioapatite of our moose samples was -14.9‰ (±1.1‰) and -16.2‰ (±1.1‰) for antlers and bones, respectively. These values are typical of C_3_ plants feeders, in agreement with the dominance of C_3_ plants in the temperate and boreal environments of Europe [21, 100, 104, 141]. Since in these areas it is not possible to determine the type of plant consumed by herbivores (i.e., to discriminate between C_3_ and C_4_ plants with different photosynthetic pathways), the small differences in the δ^13^C become crucial in identifying differences in C_3_ plant-based diet due to climatic and environmental factors [68, 168], helping to obtain a more complete picture of moose ecology.

Interestingly, our antler samples display higher δ^13^C values than bones. The first possible explanation for this difference can be related to seasonal variation in diet. Similar results were obtained by Kielland [67] for North American elk (*Alces alces gigas*) hooves, where winter values were ^13^C-depleted compared to summer. Similarly, Walter and Leslie [169] found a relative increase of δ^13^C values in the summer-portion of elk hooves (*Cervus elaphus*) from the Rocky Mountains and they interpreted this enrichment as a stronger reliance on C_4_ grasses compared to winter. This, however, is unlikely for Sweden considering that the proportion of C_4_ plants is close to 0% of the total plant species [141, 170, 171]. Moose are selective browsers feeding on a wide variety of plant species, with geographic and (intra)seasonal differences [41, 158, 172]. The foraging behaviour has been shown to depend both on food availability (which influences selectivity) and nutritional requirements, two aspects also influencing spring and fall migrations [48, 173–177]. In spring and summer, during the growing season of both plants and antlers, moose can rely on more high-quality food than in winter. The main diet components are shoots and leaves of young trees and shrubs, but also herbs, grasses and aquatic plants when available [158, 172, 178]. Preferred species are deciduous trees such as willow (*Salix* spp.), birches (*Betula* spp.), aspen (*Populus tremula*) and rowan (*Sorbus aucuparia*), but also ground vegetation released from snow such as blueberry (*Vaccinum myrtillus*) [163, 175, 176, 179]. During winter (the dormant period) moose preferentially feed on twigs and bark of the same deciduous browse species (lignin is ∼3‰ ^13^C-depleted compared to leaves, [180]), but also rely on conifers, especially the widely distributed Scots pine (*Pinus sylvestris*) [42, 50, 61, 63, 173, 177, 179, 181].

The “Canopy effect” [102, 103] could also have contributed to δ^13^C values in our samples, which is known to be detectable in the δ^13^C values of large herbivores dwelling in forested environments of boreal and temperate ecosystems [182]. Forest cover has been found to be the main factor influencing the wide variability of δ^13^C in modern moose samples from Europe [68]. Our data do not support the canopy effect as the main driving factor for differences in δ^13^C values between bones and antlers, and in general to the observed δ^13^C variability; yet, we need to stress that the moose samples analysed in this study date between the 1800 and 1994, so changes in forest cover through time might have biased our estimations. Moreover, an increased forest cover during warm seasons would have shown an inverse pattern in δ^13^C values, i.e., antlers depleted compared to bones. In terms of moose ethology, seeking forest cover is documented throughout the year to find shelter from both predators (including humans) and environmental conditions. During winter moose seek forest cover to find shelter from wind and snow (which in open environments limits access to forage). During summer they tend to avoid open habitats and use areas with dense canopies to cope with high temperatures, especially when above 20°C [158, 183, 184].

Overall, the differences in δ^13^C between moose bones and antlers likely result from a combination of factors. Moose display a complex foraging behaviour that changes seasonally according to mobility, nutritional requirements, food availability and environmental factors [61, 63, 150, 158, 173, 175]. Besides, we cannot exclude eventual sub-annual fluctuations in atmospheric CO_2_ isotope composition as a possible driving factor for δ^13^C in moose tissues [185]. In addition, the consumption of lipids may shift the δ^13^C values of tissues toward more negative values, e.g., during periods of metabolic stress. Assuming possible winter stress of moose due to limited food resources, lower δ^13^C values in year-round remodelling bones compared to spring-summer growing antlers and can be partially explained by an increased catabolism of endogenous fat reserves during the winter season (see e.g., [186]).

## 6. Conclusion and future perspectives

We report here a dataset of strontium (^87^Sr/^86^Sr), oxygen (δ^18^O) and carbon (δ^13^C) isotope values of modern moose bones and antlers from Sweden. This is the largest database of moose isotope values presented so far for the area and can be used for future research in *Alces* sp. (palaeo)ecology and in general as a modern reference dataset for mammal isotope compositions of Scandinavia. However, our samples and their metadata come from individuals collected during the 19th century and, as such, we cannot be fully confident in their geographic assignment and dating. This is a common problem when dealing with samples from historic collections (see e.g. [187]). Nevertheless, most of the moose samples here considered are compatible with the local predicted isotope baseline, reinforcing the hypothesis of a correct geographic assignment.

We compiled bioavailable Sr isotope data from literature and used them to build the first Sr isoscape of whole Scandinavia using a machine learning approach. Even if additional bioavailable Sr isotope samples will further improve its predictive power, the Sr isoscape presented herein can be a useful tool for provenance studies in the area (both for modern and archaeological faunal or human samples, but also for foods, artefacts and plant materials).

After comparison with the constructed isoscapes, the seasonal (antler) and multi-annual (bone) mobility assessed through the workflow presented in this paper agrees well with ecological data on moose movement behaviour (i.e., home-range, migration). Even if there are some limiting factors to our interpretation (i.e., errors associated with the isoscape in S6 Fig and a likely biased representation of moose diet in the isoscape, see Results), the Bayesian workflow presented here will provide a better understanding of how moose move across the Scandinavian landscape, and become a useful mean to support traditional mobility tracking methods (e.g., radio-, satellite- and GPS-tracking) and genetic analyses. Future work combining animal tracking with isotope analysis will advance our understanding of the relationship between isotope values and animal behaviour.

The δ^13^C values are typical of C_3_ plant feeders, and the differences detected between antlers (^13^C-enriched) and bones (^13^C-depleted) are likely due to seasonal differences in dietary habits and/or physiological stress during winter. Sub-annual fluctuations of atmospheric CO_2_ levels may also have affected the δ^13^C values of moose. However, further work is needed (i.e., high-resolution sampling/analyses) to precisely understand winter-summer isotopic differences in moose hard tissues to interpret their diet and physiology in a comprehensive way.

In our view, especially in light of the growing concern about the effects of climate change on animal migration and biodiversity conservation, geochemical analyses should become an integral part of multidisciplinary studies aimed at reconstructing wildlife mobility.

## Supporting information

S1 Fig (A) Calculated intra-site (i.e. same coordinates’ samples) standard deviations for literature values used in building the isoscape vs. their mean isotope values. (B) Extrapolated RF error (from the error map) at the same sites vs. the mean isotope values as in A. (C) Calculated intra-site standard deviations for literature values vs. extrapolated RF error. See Materials and Methods for discussion.
(DOCX)

S2 Fig Random forest model performances. (A) Modelled Sr isotope ratios vs observed Sr isotope ratios of isoscape samples; the 10-fold cross-validation resulted in an RMSE = 0.0055 and an R^2^ = 0.65. (B) Variable importance of the seven external predictors (r.srsrq3, r.fert, r.elevation, r.ssa, r.cec, r.ssaw and r.mat) selected by using the VSURF algorithm. See Bataille et al. (2020) for details. (C) Partial dependence plot for the VSURF-selected variables.
(DOCX)

S3 Fig Δ^87^Sr/^86^Sr_sample-isoscape_ and Δ^18^O_sample-isoscape_ plotted over the Scandinavia map. (DOCX)

S4 Fig Δ^87^Sr/^86^Sr_sample-isoscape_ and Δ^18^O_sample-isoscape_ plotted vs individual sex. Only samples of known sex were considered.
(DOCX)

S5 Fig Difference between calibrated-uncalibrated Sr (A) and O (B) isoscapes. Calibrated isoscapes were obtained by using Δs of moose samples within 1σ (see the main text for details).
(DOCX)

S6 Fig Sr isoscape (RF model) error map. The calculation was carried out as reported in the Materials and Methods section of the main text.
(DOCX)

S1 Table Moose samples’ data. (XLSX)

S2 Table Literature values used to build the Scandinavia isoscape. (XLSX)

S3 Table Coefficients of determination of sample vs isoscape isotope ratio regression. P values are always << 0.01.
(XLSX)

S1 File Sr isoscape of Scandinavia as GeoTIFF. (TIF)

S2 File Sr uncertainty map of Scandinavia as GeoTIFF. (TIF)
