# Supplementary material for: Spatial ecology of moose in Sweden: Combined Sr-O-C isotope analyses of bone and antler
Source: PLoS One. 2024 Apr 10;19(4):e0300867. doi: 10.1371/journal.pone.0300867 (PMC11006136; doi:10.1371/journal.pone.0300867)
Supplement: S1 Fig — (A) Calculated intra-site (i.e. same coordinates’ samples) standard deviations for literature values used in building the isoscape vs. their mean isotope values. (B) Extrapolated RF error (from the error map) at the same sites vs. the mean isotope values as in A. (C) Calculated intra-site standard deviations for literature values vs. extrapolated RF error. See Materials and Methods for discussion. (DOCX) [file pone.0300867.s001.docx]

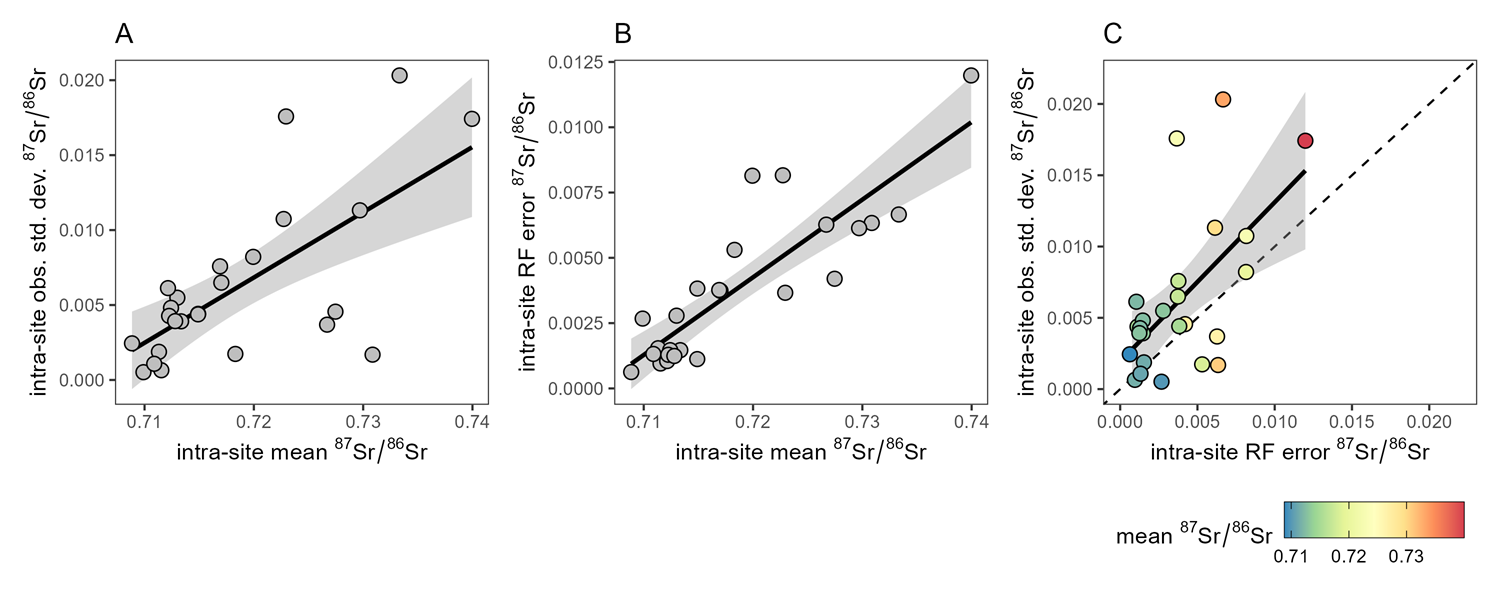
 **S1_fig.** (A) Calculated intra-site (i.e. same coordinates’ samples) standard deviations for literature values used in building the isoscape vs. their mean isotope values. (B) Extrapolated RF error (from the error map) at the same sites vs. the mean isotope values as in A. (C) Calculated intra-site standard deviations for literature values vs. extrapolated RF error. See Materials and Methods for discussion.
