# Supplementary material for: Spatial ecology of moose in Sweden: Combined Sr-O-C isotope analyses of bone and antler
Source: PLoS One. 2024 Apr 10;19(4):e0300867. doi: 10.1371/journal.pone.0300867 (PMC11006136; doi:10.1371/journal.pone.0300867)
Supplement: S2 Fig — (A) Modelled Sr isotope ratios vs observed Sr isotope ratios of isoscape samples; the 10-fold cross-validation resulted in an RMSE = 0.0055 and an R2 = 0.65. (B) Variable importance of the seven external predictors (r.srsrq3, r.fert, r.elevation, r.ssa, r.cec, r.ssaw and r.mat) selected by using the VSURF algorithm. See Bataille et al. (2020) for details. (C) Partial dependence plot for the VSURF-selected variables. (DOCX) [file pone.0300867.s002.docx]

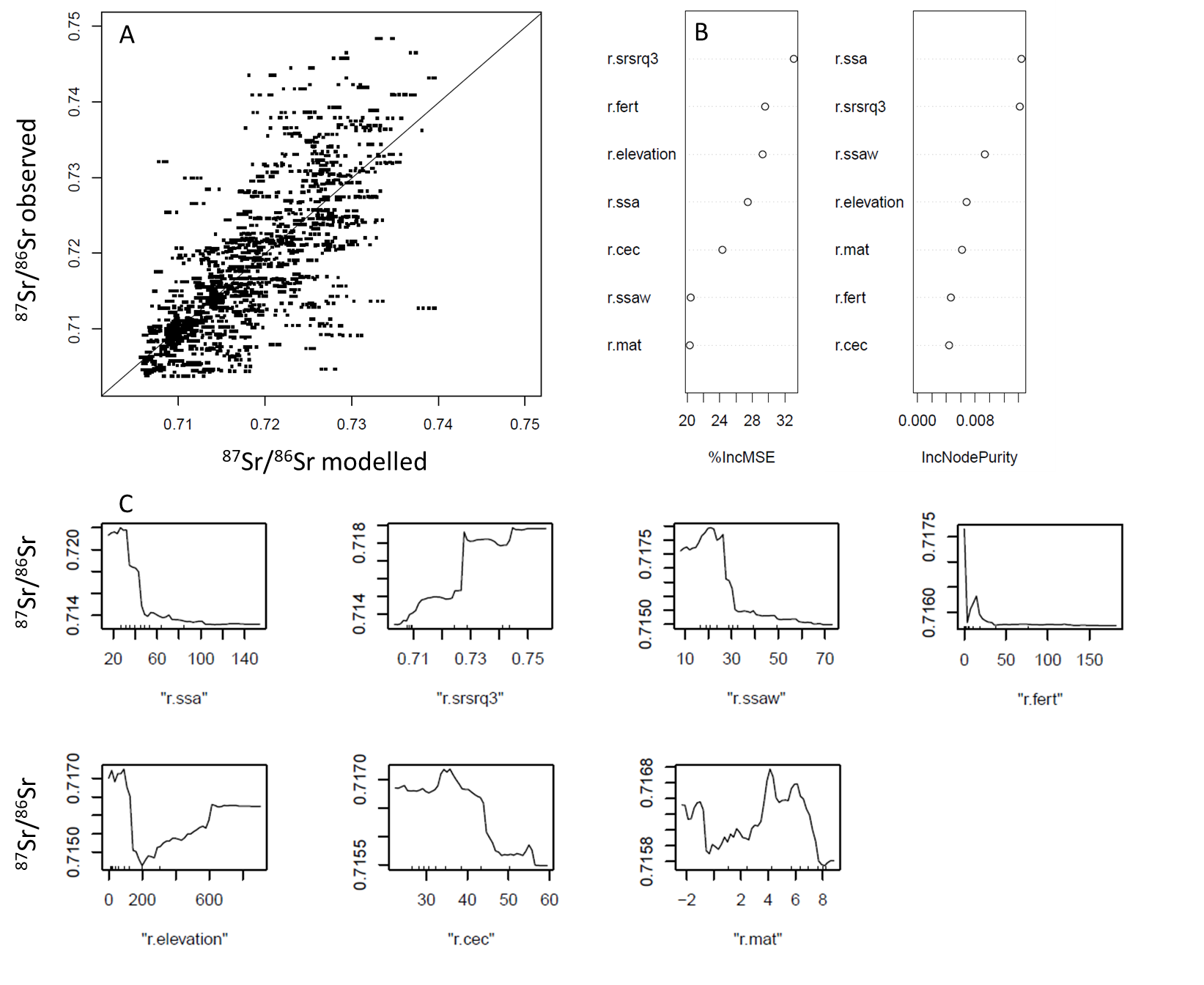


**S2_fig. Random forest model performances.** (A) Modelled Sr isotope ratios vs observed Sr isotope ratios of isoscape samples; the 10-fold cross-validation resulted in an RMSE = 0.0055 and an R^2^ = 0.65. (B) Variable importance of the seven external predictors (r.srsrq3, r.fert, r.elevation, r.ssa, r.cec, r.ssaw and r.mat) selected by using the VSURF algorithm. See Bataille et al. (2020) for details. (C) Partial dependence plot for the VSURF-selected variables.
