# Supplementary figures and images for: Spatial ecology of moose in Sweden: Combined Sr-O-C isotope analyses of bone and antler
Source: PLoS One. 2024 Apr 10;19(4):e0300867. doi: 10.1371/journal.pone.0300867 (PMC11006136; doi:10.1371/journal.pone.0300867)

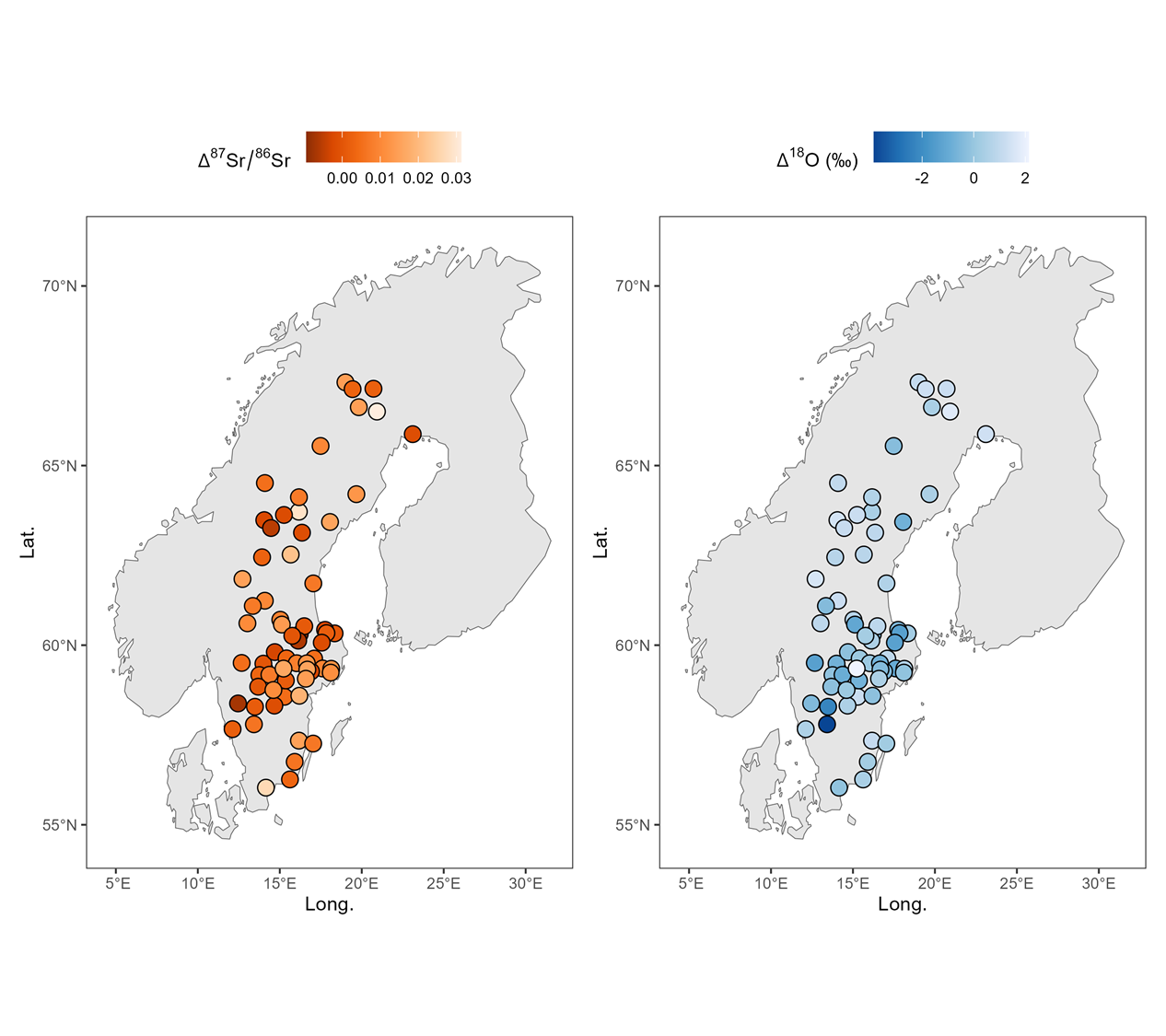


**S3_fig. Δ87Sr/86Srsample-isoscape and Δ18Osample-isoscape plotted over the Scandinavia map.**

Supplement: S3 Fig — (DOCX) [file pone.0300867.s003.docx]
