# Supplementary material for: Spatial ecology of moose in Sweden: Combined Sr-O-C isotope analyses of bone and antler
Source: PLoS One. 2024 Apr 10;19(4):e0300867. doi: 10.1371/journal.pone.0300867 (PMC11006136; doi:10.1371/journal.pone.0300867)
Supplement: S4 Fig — Only samples of known sex were considered. (DOCX) [file pone.0300867.s004.docx]

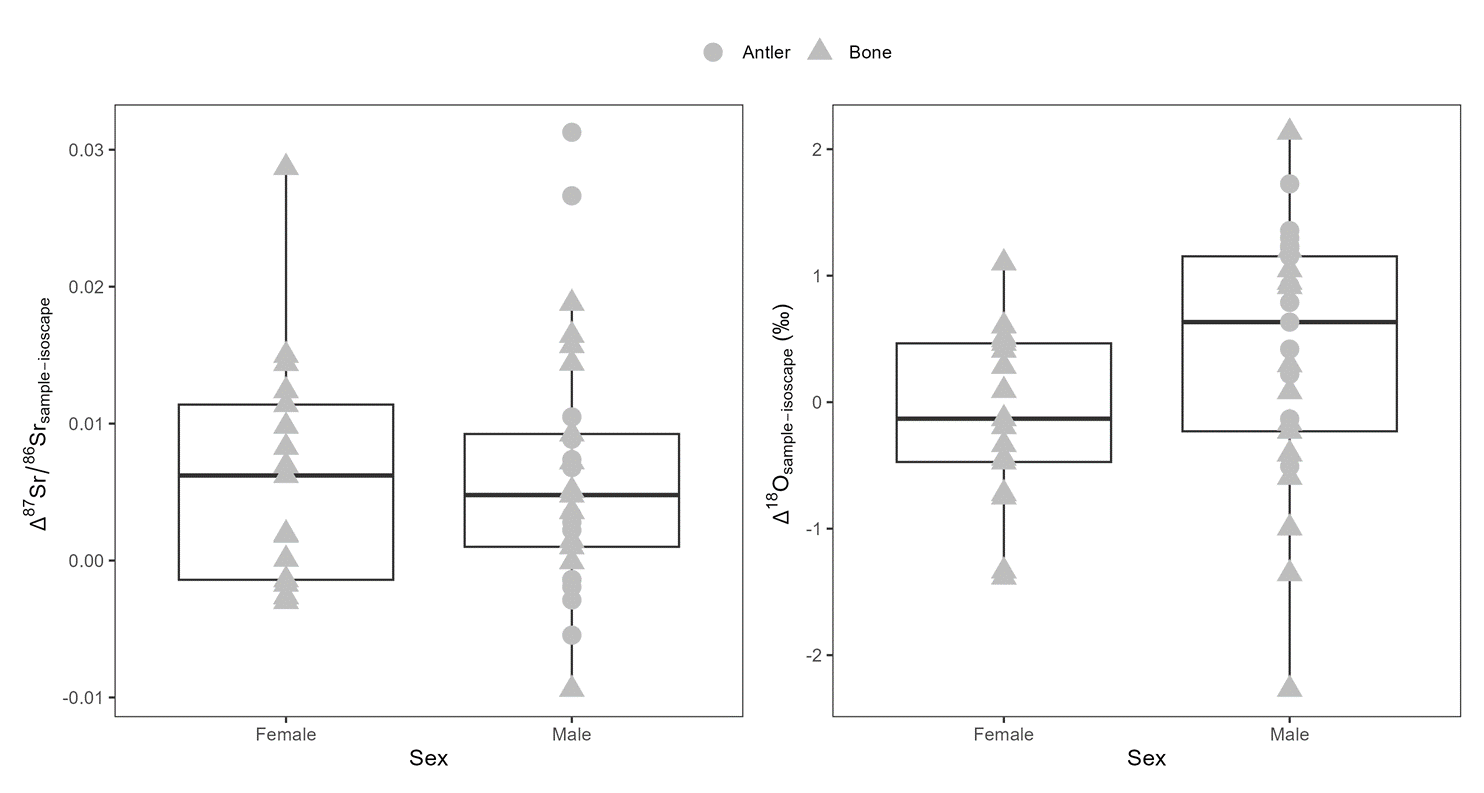


**S4_fig.** **Δ^87^Sr/^86^Sr_sample-isoscape_ and Δ^18^O_sample-isoscape_ plotted vs individual sex.** Only samples of known sex were considered.
