# Supplementary material for: Spatial ecology of moose in Sweden: Combined Sr-O-C isotope analyses of bone and antler
Source: PLoS One. 2024 Apr 10;19(4):e0300867. doi: 10.1371/journal.pone.0300867 (PMC11006136; doi:10.1371/journal.pone.0300867)
Supplement: S5 Fig — Difference between calibrated-uncalibrated Sr (A) and O (B) isoscapes. Calibrated isoscapes were obtained by using Δs of moose samples within 1σ (see the main text for details). (DOCX) [file pone.0300867.s005.docx]

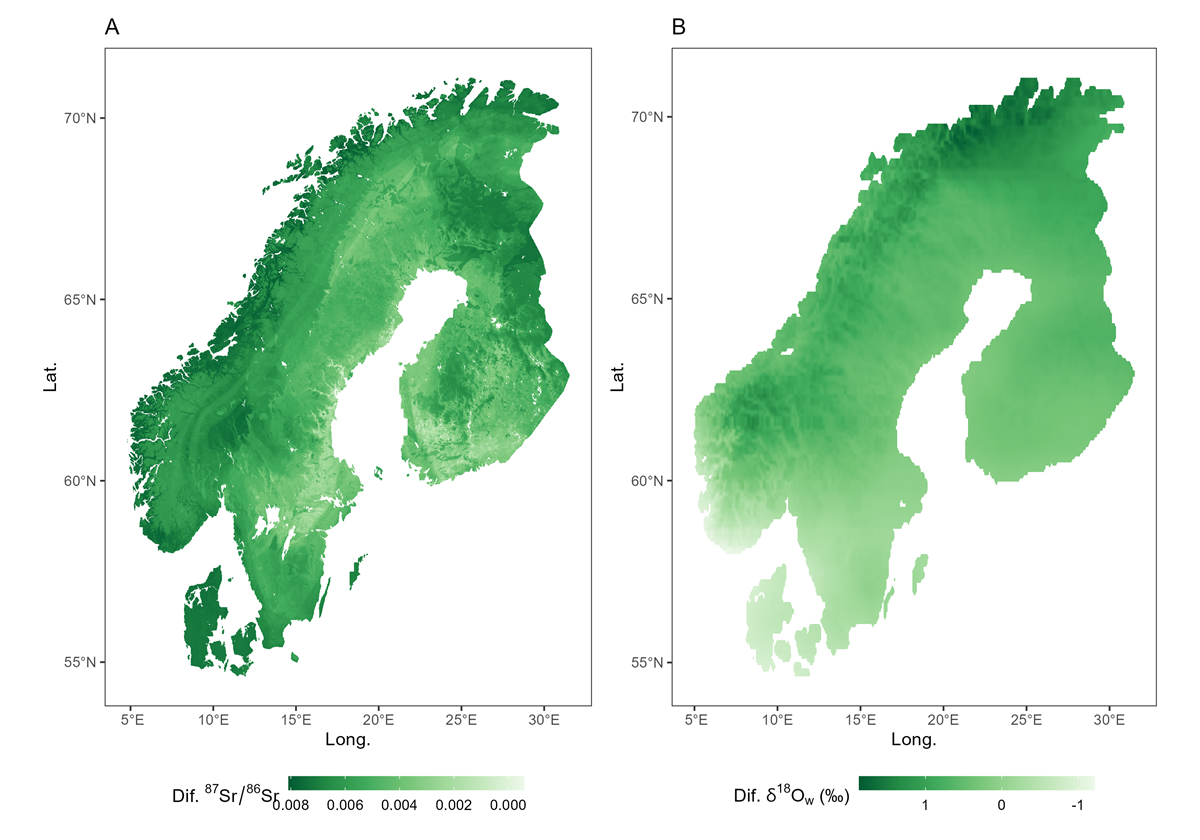


**S5_fig. Difference between calibrated-uncalibrated Sr (A) and O (B) isoscapes.** Calibrated isoscapes were obtained by using Δs of moose samples within 1σ (see the main text for details).
