# Supplementary material for: Spatial ecology of moose in Sweden: Combined Sr-O-C isotope analyses of bone and antler
Source: PLoS One. 2024 Apr 10;19(4):e0300867. doi: 10.1371/journal.pone.0300867 (PMC11006136; doi:10.1371/journal.pone.0300867)
Supplement: S6 Fig — The calculation was carried out as reported in the Materials and Methods section of the main text. (DOCX) [file pone.0300867.s006.docx]

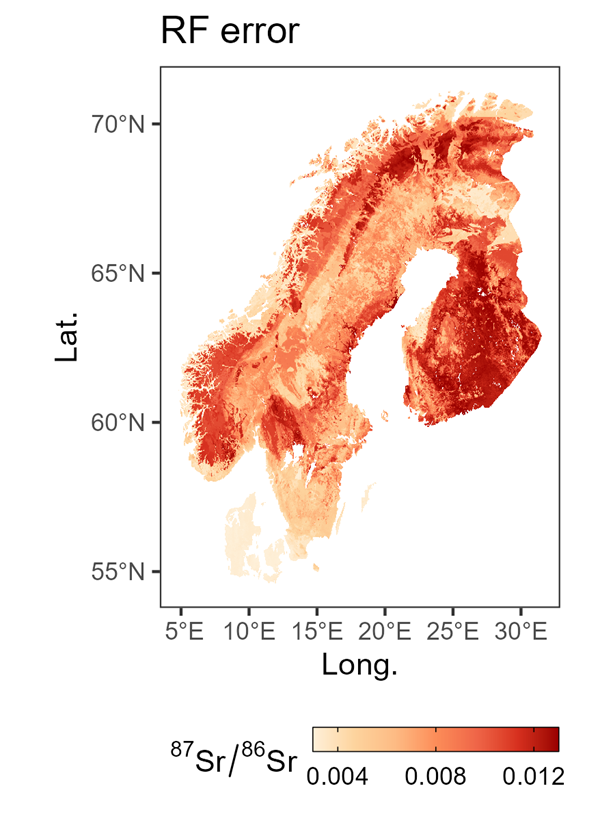


**S6_fig. Sr isoscape (RF model) error map.** The calculation was carried out as reported in the Materials and Methods section of the main text.
